# Factors Associated With Delayed Gastric Emptying in Symptomatic Diabetic and Non-diabetic Patients: A Retrospective Observational Study

**DOI:** 10.7759/cureus.58038

**Published:** 2024-04-11

**Authors:** Mostafa Shehata, Ibrahim Al Hosani, Yashbir Singh, Ameirah Alali, Shaima Khan, Mohamed Al Zaabi, Omar Khadam, Maryam Alahmad, Rizwan Syed, Khalifa Al Tiniji, Abdulla Aljanahi, Eyad Al Akrad

**Affiliations:** 1 Gastroenterology, Sheikh Shakhbout Medical City, Abu Dhabi, ARE; 2 Department of Radiology, Mayo Clinic, Rochester, USA; 3 Gastroenterology and Hepatology, Sheikh Shakhbout Medical City, Abu Dhabi, ARE; 4 Internal Medicine, Sheikh Shakhbout Medical City, Abu Dhabi, ARE; 5 Radiology, Sheikh Shakhbout Medical City, Abu Dhabi, ARE

**Keywords:** delayed gastric emptying, predictors of gastroparesis, gastric emptying study, nausea and vomiting, diabetic gastroparesis (dg)

## Abstract

Background

Gastroparesis, characterized by delayed gastric emptying without mechanical obstruction, is a significant complication, especially in diabetic individuals. It manifests through symptoms such as abdominal bloating, feelings of fullness, and pain. This study investigates the prevalence of gastroparesis among non-diabetic and diabetic patients, exploring associations with demographic data, hemoglobin A1C (HbA1C) levels, and symptoms.

Methodology

This retrospective, observational, cohort study included patients with gastroparesis symptoms who underwent a nuclear gastric emptying study from January 2021 to April 2023. The study analyzed demographic data, symptoms, and HbA1c levels to identify correlations with delayed gastric emptying.

Results

Of 157 patients, 34.4% exhibited delayed gastric emptying. Diabetic patients comprised 29.3% of the sample, with a notable disease duration of over 10 years in 77.3% of cases. Symptoms such as nausea, vomiting, epigastric pain, and early satiety were prevalent, with significant associations between delayed emptying and female gender, higher HbA1c, and vomiting.

Conclusions

Delayed gastric emptying is significantly associated with female gender, elevated HbA1c levels, and when vomiting is the presenting symptom. Highlighting the importance of awareness among healthcare providers and the community, the findings encourage collaborative efforts for further gastroparesis research to better understand the predictive factors and mechanisms.

## Introduction

Gastroparesis, a condition characterized by delayed gastric emptying without mechanical obstruction, is commonly seen as a significant complication of diabetes mellitus [1,2]. Symptoms such as abdominal bloating, feelings of fullness, and upper abdominal pain are often linked with this delay in gastric emptying among diabetic individuals [3-6]. Radionuclide imaging for gastric emptying is widely recognized as the definitive method for assessing patients with suspected gastric emptying disorders [7]. Since radionuclide scintigraphy’s initial application in 1966 for evaluating gastric emptying, it has been established as a valuable, non-invasive technique for quantitatively analyzing gastric emptying rates [8]. This method involves the ingestion of a meal labeled with a radionuclide, allowing for the measurement of gastric activity as an indicator of gastric disorders [9]. Studies utilizing radionuclide scintigraphy have reported the prevalence of delayed gastric emptying to be between 25% and 55% in patients with type 1 diabetes and 30% in those with type 2 diabetes [10,11]. It has also been found that 29% of individuals diagnosed with gastroparesis are diabetic [12]. Various factors in diabetic patients can influence gastric emptying times, which should be considered when interpreting results. For instance, acute changes in blood glucose levels can affect the gastric emptying rate of both liquids and solids [13]. While gastroparesis has often been linked to uncontrolled hyperglycemia, some studies have found no direct correlation between gastroparesis and autonomic dysfunction [4,11,14]. However, some studies have observed associations between diabetic gastroparesis, cardiovascular disease, and retinopathy [15]. The Society of Nuclear Medicine and Molecular Imaging and the American Society of Neurogastroenterology and Motility have endorsed a standard diet and imaging protocol for gastric emptying evaluations based on a study involving 123 healthy participants [16]. Despite extensive research on gastric emptying assessment through scintigraphy, some aspects remain debated. In studies investigating the diagnosis of gastroparesis, endoscopic findings of food remnants in the stomach, despite expected scintigraphy results, suggest gastroparesis in the absence of gastric outlet obstruction [17,18]. However, the 2014 Diabetes Care Standards did not include endoscopy as a diagnostic tool for gastroparesis [17,18]. There are indications that a subset of patients, particularly those experiencing rapid gastric emptying accompanied by nausea, bloating, and fullness, might benefit from scintigraphic gastric emptying evaluation [19-22]. This phenomenon has been similarly observed in patients with functional dyspepsia and may be an early sign of type 2 diabetes in some instances. Symptoms often overlap with those of gastroparesis, and rapid gastric emptying has been reported to be more prevalent than delayed emptying in patients with autonomic dysfunction.

The study aims to explore the prevalence of gastroparesis among symptomatic non-diabetic and diabetic patients, as well as the associations of gastric emptying with demographic data, hemoglobin A1c (HbA1c), and various symptoms of gastroparesis.

## Materials and methods

Study setting and design

This study was conducted as a retrospective, observational, cohort study at Sheikh Shakhbout Medical City, a tertiary care referral hospital in the United Arab Emirates. The study protocol received approval from the Institutional Review Board (IRB) (approval number: SSMCREC-404), ensuring compliance with ethical guidelines.

Participants

The cohort included patients aged 16 years and above who underwent a nuclear gastric emptying study (GES) from January 1, 2021, to April 30, 2023. Inclusion criteria were symptomatic patients confirmed to have no obstruction via upper gastrointestinal endoscopy or imaging. Exclusion criteria encompassed asymptomatic patients, those with inconclusive or rapid gastric emptying results, individuals with previous gastric surgery, and patients already diagnosed and treated for gastroparesis.

Data collection

Medical records were retrospectively reviewed, and relevant data were extracted from electronic health records post-IRB approval. The collected data included age, gender, body mass index (BMI), diabetes status, duration of diabetes, HbA1c levels within two months of the study, and symptoms such as nausea, vomiting, early satiety, and epigastric pain. All patient data were anonymized and securely stored for analysis. Logistic regression analysis was conducted using univariate and multivariate models to explore associations between studied variables and delayed gastric emptying.

Gastric emptying study

Patient Preparation

Patients of childbearing potential were screened for pregnancy and lactation status and advised on the timing of their menstrual cycle to minimize hormonal effects on gastrointestinal motility. Prokinetic and gastric emptying-delaying medications were discontinued 72 hours before testing [23,24].

Examination Protocol

The study duration was up to five hours, utilizing a 0.5 mCi dose of 99mTc-nanocolloid. The meal for the test consisted of liquid egg whites mixed with the radiopharmaceutical, toasted white bread with jam, and water. Imaging was scheduled at zero, one, two, three, and four hours post-meal, with an additional dynamic scan early if gastroesophageal reflux was a concern.

Imaging Equipment and Procedure

A gamma camera with a low-energy general-purpose collimator was used, setting an energy window at 140 keV. The patient was positioned supine with the imaging field covering the upper abdomen. The imaging protocol outlined specific settings for static and dynamic acquisitions, ensuring standardized data collection.

This comprehensive methodology provided a structured framework for investigating the association between various factors and delayed gastric emptying, utilizing a detailed approach to patient selection, data collection, and imaging protocol to ensure the reliability and validity of the findings [25]. If imaging shows that more than 10% of the tracer remains in the stomach at one, two, or three hours, recent literature cites the need to obtain images for up to four hors, suggesting that retention of more than 10% of the meal in the stomach at four hours is abnormal and the best discriminator between normal and abnormal results (Table 1).

**Table 1:** Normal limits for gastric retention.

Time point	Upper limit (a greater value suggests abnormally delayed gastric emptying)
1.0 hour	90%
2.0 hour	60%
3.0 hour	30%
4.0 hour	10%

Statistical analysis

Data were entered into the computer using the SPSS version (IBM Corp., Armonk, NY, USA). Data were entered as numerical or categorical, as appropriate. Two types of statistics were done: (i) Descriptive statistics: Qualitative data were expressed as frequency and percentage. Quantitative data were shown as mean, SD, median, range (minimum-maximum), and interquartile range. (ii) Analytical statistics: A chi-square test was used to measure the association between qualitative variables. The Mann-Whitney test was used to compare two sets of quantitative, not normally distributed data. Logistic regression model was used to give an adjusted odds ratio (OR) and 95% confidence interval (CI) of the effect of the different risk factors for the subject under the study. The p-value was considered statistically significant at less than 0.05.

## Results

A total of 157 patients were included in the study; ages ranged from 17 years to 88 years, and the median age was 41 years. Of these, 53 (33.8%) were male, and 104 (66.2%) were female. The gastric emptying study was normal in 103 (65.6%) patients and showed delayed emptying in 54 (34.4%) patients. Overall, 46 patients were diabetic, accounting for 29.3% of the cohort, while 111 (70.7%) patients did not have diabetes at the time of the study. Most diabetic patients had a disease duration of more than 10 (77.3%) years. HbA1c levels were available within two months from the study for 85 patients and ranged from 0.5 to 14 mg/dL, with a mean of 6.26 mg/dL. Nausea was the presenting symptom in 106 (67.5%) patients, while vomiting was present in 59 (37.6%) patients. Epigastric pain was a symptom in 126 (80.3%) patients, and early satiety was reported in 63 (40.1%) patients. Most patients presented with a combination of two or three symptoms, constituting 70% of the entire cohort. In contrast, 38 (24%) patients presented with only one symptom, and nine (6%) patients presented with a combination of the four symptoms of gastroparesis (Table 2).

**Table 2:** Characteristics data of patient (delayed and normal) (n = 157). IQR = interquartile range (Q1-Q3); BMI = body mass index; DM = diabetes mellitus; HbA1C = hemoglobin A1c

Characteristics		Value	Percent
Age (years)	Mean ±SD	45.18 ± 19.21	
	Median (IQR)	41 (28–62)	
	Minimum–maximum	17–88	
Gender	Male	53	33.8
	Female	104	66.2
Nationality	Emirate	117	74.5
	Non-Emirate	40	25.5
BMI (kg/m^2^)	Less than 18	8	5.1
	18–24	52	33.1
	25–30	39	24.8
	30–35	28	17.8
	35–40	22	14.0
	More than 40	8	5.1
Gastric emptying	Normal	103	65.6
	Delayed	54	34.4
DM	Yes	46	29.3
	No	111	70.7
Duration DM (n = 44) (years)	Less than 10	10	22.7
	More than 10	37	77.3
HbA1C (n = 85)	Mean ± SD	6.26 ± 1.87	
	Median (IQR)	5.9 (5.3–7.3)	
	Minimum–maximum	0.1–14	
Nausea	Yes	106	67.5
	No	51	32.85
Vomiting	Yes	59	37.6
	No	98	62.4
Epigastric pain	Yes	126	80.3
	No	31	19.7
Early satiety	Yes	63	40.1
	No	94	59.9
Symptoms (n)	1	38	24.2
	2	52	33.1
	3	58	36.9
	4	9	5.7
Total		157	100

A comparison between patients with delayed gastric emptying and patients with normal gastric emptying study using univariate data analysis showed that delayed gastric emptying is associated with female gender (p = 0.01) and when the patient presented with vomiting (p = 0.048). There was no significant difference regarding the other variables, including diabetes mellitus, BMI, and age (Table 3).

**Table 3:** Univariate data analysis. Comparison between patients with delayed gastric emptying and those with normal gastric emptying. χ^2^: chi-square test; U: Mann-Whitney U test; *: significant. IQR = interquartile range (Q1-Q3); BMI = body mass index; DM = diabetes mellitus; HbA1C = hemoglobin A1c

		Gastric emptying		Test of sig	P-value
		Delayed	Normal		
Age (years)	N	53	103	U = 0.133	0.894
	Mean ± SD	44.98 ± 20.158	45.28 ± 18.8		
	Median (IQR)	41 (26-61.5)	43 (29-62)		
	Minimum–maximum	17-87	18-88		
Gender	Male	11 (20.4%)	42 (40.8%)	χ^2^ = 6.59	0.01*
	Female	43 (79.6%)	61 (59.2%)		
Nationality	Emirate	39 (72.2%)	78 (75.7%)	χ^2^ = 0229	0.632
	Non-Emirate	15 (27.8%)	25 (24.3%)		
BMI (kg/m^2^)	Less than 18	4 (7.4%)	4 (3.9%)	χ^2^ = 1.99	0.85
	18–25	20 (37%)	32 (31.1%)		
	25–30	12 (22.2%)	27 (26.2%)		
	30–35	8 (14.8%)	20 (19.4%)		
	35–40	7 (13%)	15 (14.6%)		
	More than 40	3 (5.6%)	5 (4.9%)		
DM	Yes	14 (25.9%)	29 (28.2%)	χ^2^ = 0.089	0.766
	No	40 (74.1%)	74 (71.8%)		
Duration DM (n = 44) (years)	Less than 10	3 (20%)	7 (24.1%)	χ^2^ = 0.096	0.756
	More than 10	12 (80%)	22 (75.9%)		
HbA1C (n = 85)	Mean ± SD	6.727 ± 20.1	6.006 ± 2.033	U = 0.792	0.429
	n	30	55		
	Median (IQR)	5.9 (5.275–7.85)	5.9 (5.3–6.7)		
	Minimum–maximum	4.4–14	0.1–11.9		
Nausea	Yes	37 (68.5%)	69 (67%)	χ^2^ = 0.038	0.846
	No	17 (31.5%)	34 (33%)		
Vomiting	Yes	26 (48.1%)	33 (32%)	χ^2^ = 3.919	0.048*
	No	28 (51.9%)	70 (68%)		
Epigastric pain	Yes	41 (75.9%)	85 (82.5%)	χ^2^ = 0.973	0.324
	No	13 (24.1%)	18 (17.5%)		
Early satiety	Yes	23 (42.6%)	40 (38.8%)	χ^2^ = 0.208	0.648
	No	31 (57.4%)	63 (61.2%)		
Symptoms (n)	1	12 (22.2%)	26 (25.2%)	χ^2^ = 2.1	0.552
	2	15 (27.8%)	37 (35.9%)		
	3	24 (44.4%)	34 (33%)		
	4	3 (5.6%)	6 (5.8%)		

Table 4 shows the multivariate logistic regression model I (variables entered were age, gender, diabetes, vomiting, epigastric pain, and early satiety). The most essential significant variables were female gender and vomiting. In the study, female participants were more likely to have delayed gastric emptying than males (p = 0.010). The odds of delayed gastric emptying among study participants or cases of vomiting were about 2.113 times the odds for those who did not experience vomiting (OR = 2.113, 95% CI = 1.003-4.450, p = 0.049) (Table 4).

**Table 4:** Multivariate logistic regression model I analysis. Variables entered: age, gender, DM, vomiting, epigastric pain, and early satiety. *: significant p-value. DM = diabetes mellitus; CI = confidence interval

	Exp(B)	95% CI for Exp (B)		P-value
		Lower	Upper	
Age	0.996	0.976	1.017	0.707
Gender (female) (reference: male)	2.873	1.283	6.431	0.010*
DM	0.769	0.312	1.899	0.569
Vomiting	2.113	1.003	4.450	0.049*
Epigastric pain	0.696	0.293	1.656	0.413
Early satiety	1.409	0.662	3.000	0.373
Constant	0.390			0.471

Table 5 shows the multivariate logistic regression model II (variables entered were age, gender, HbA1C, nausea, vomiting, epigastric pain, and early satiety). The most essential significant variables were female gender and HbA1C. The odds of having delayed gastric emptying among female participants were about 5.456 times the odds for males (OR = 5.456, 95% CI = 1.526-19.510, p = 0.009). The odds of having delayed gastric emptying were 1.425, with each 1% increase in HbA1C (OR = 1.425, 95% CI = 1.526-1.946, p = 0.026).

**Table 5:** Multivariate logistic regression model II analysis. Variables entered: age, gender, HbA1c, nausea, vomiting, epigastric pain, and early Satiety. Key significant variables: female gender and HbA1c. HbA1C = hemoglobin A1C; CI = confidence interval

	Exp(B)	95% CI for Exp (B)		P-value
		Lower	Upper	
Age	0.988	0.961	1.014	0.362
Gender (female) (reference: male)	5.456	1.526	19.510	0.009*
HbA1C	1.425	1.043	1.946	0.026*
Nausea	0.800	0.229	2.798	0.727
Vomiting	3.183	0.892	11.362	0.075
Epigastric pain	0.953	0.212	4.296	0.950
Early satiety	2.298	0.712	7.412	0.164
Constant	0.019			0.016

## Discussion

Although the role of delayed gastric emptying as a core factor in defining gastroparesis has been debated, the current definition still requires the presence of this delay in addition to specific gastrointestinal symptoms and the absence of gastric outlet obstruction [26,27]. This study aims to establish the prevalence, characteristics, and risk factors of gastroparesis. This retrospective study demonstrated findings that were, to a certain extent, similar to those of other international studies, such as the higher prevalence in women. Many studies have attempted to identify risk factors for delayed gastric emptying, focusing on specific post-surgical patient groups, such as those who underwent pancreaticoduodenectomy, gastrectomy, or esophagostomy [28-31]. Delayed gastric emptying was associated with female gender and vomiting, among other intra and postoperative factors. In our study, patients presented with symptoms suspicious of gastroparesis without any perioperative history. In univariate analysis, delayed gastric emptying was strongly associated with female gender and vomiting. Unlike many other non-surgical studies, BMI, other gastroparesis-related symptoms, the number of symptoms, diabetes mellitus, years of being diabetic, and HbA1c did not predict delayed gastric emptying [32-35]. Surprisingly, in our study, multivariate analysis showed an association of delayed gastric emptying with increased HbA1c levels. As we excluded asymptomatic patients from this study, these associations can also apply to gastroparesis.

In a similar study, multivariate analysis also found a link between delayed gastric emptying and higher HbA1c levels, indicating a potential common pathophysiological mechanism in patients with symptoms of gastroparesis. This further supports the notion that glycemic control may play a significant role in managing gastroparesis symptoms.

The limitations of this study include its small size and the fact that the data came from a single center. The design of this study fell short of establishing the epidemiology of gastroparesis in the UAE. Nevertheless, this is the first study in the region utilizing nuclear medicine gastric emptying study to confirm the diagnosis, answer some questions regarding the disease characteristics, and compare them to those of other international cohorts. For example, one-third of the 157 patients who presented with symptoms suggestive of gastroparesis were confirmed to have the disease. Almost one-third of the study sample had diabetes mellitus, and around 25% met the criteria for gastroparesis. In our cohort, nausea was the most common presenting symptom; however, vomiting was the only symptom to show a statistically significant association. Interestingly, this study revealed that the risk of delayed gastric emptying was one and a half times higher for every 1% increase in HbA1c.

## Conclusions

Our study showed that almost one-third of patients presented with the common upper gastrointestinal symptoms will have delayed gastric emptying. The factors associated and predictive of delayed gastric emptying were female gender and HbA1c and vomiting. This finding requires attention from healthcare providers to consider this diagnosis considering the different management from other differential diagnoses for such symptoms. Awareness of the disease among healthcare providers and the community can draw more attention and support, creating a collaborative effort for larger studies to answer more questions regarding the factors associated with and predictive of delayed gastric emptying.
